# Supplementary material for: Central nervous system infection in the intensive care unit: Development and validation of a multi-parameter diagnostic prediction tool to identify suspected patients
Source: PLoS One. 2021 Nov 29;16(11):e0260551. doi: 10.1371/journal.pone.0260551 (PMC8629274; doi:10.1371/journal.pone.0260551)
Supplement: S2 File — (DOCX) [file pone.0260551.s002.docx]

**S2 File - Supporting data for “Central nervous system infection in the intensive care unit: development of a multi-parameter diagnostic prediction tool to identify suspected patients”**

**S1 Table:** Diagnostic Criteria for Encephalitis and Encephalopathy of Presumed Infectious or Autoimmune Etiology by Venkatesan et al 2013

| **MAJOR CRITERION (required): ENCEPHALOPATHY -** Patients presenting to medical attention with altered mental status (defined as decreased or altered level of consciousness, lethargy or personality change) lasting ≥24 h with no alternative cause identified. |
| --- |
|  |
|  |
| **Minor Criteria** (2 required for possible encephalitis; ≥3 required for probable or confirmed encephalitis) ^a^ |
| **FEVER**: Documented fever ≥38° C (100.4°F) within the 72 h before or after presentation ^b^ |
| **SEIZURES**: Generalized or partial seizures not fully attributable to a preexisting seizure disorder ^c^ |
| **FOCAL SIGNS**: New onset of focal neurologic findings – hemiplegia, paraplegia, tetraplegia |
| **CEREBROSPINAL FLUID**: CSF WBC count ≥5/cubic mm ^d^ |
| **IMAGING**: Abnormality of brain parenchyma on neuroimaging suggestive of encephalitis that is either new from prior studies or appears acute in onset ^e^ |
| **EEG**: Abnormality on electroencephalography that is consistent with encephalitis and not attributable to another cause.^f^ |
| ^a^ Confirmed encephalitis requires one of the following: (1) Pathologic confirmation of brain inflammation consistent with encephalitis; (2) Defined pathologic, microbiologic, or serologic evidence of acute infection with a microorganism strongly associated with encephalitis from an appropriate clinical specimen or (3) Laboratory evidence of an autoimmune condition strongly associated with encephalitis.  ^b^ Fever is a common finding in patients with acute encephalitis but is nonspecific. The requirement for objective documentation of fever within a restricted time frame of ≤72 h after hospitalization was chosen to exclude secondary healthcare associated infections. It is recognized that fevers can occur because of several infections outside of the central nervous system that can cause encephalopathy, as well as with noninfectious entities that mimic encephalitis. It is also recognized that fever may fluctuate and, as such, objective fever may be lacking in patients with infectious encephalitis at the time of clinical assessment. Furthermore, immunosuppressed patients with encephalitis may not mount a fever.  ^c^ Seizures associated with encephalitis may be generalized, suggestive of global CNS dysfunction, or focal, indicating a localized process. Subclinical seizures may also occur and can be a cause of altered sensorium. Seizures associated with high temperatures are relatively common in young children and, if occurring in isolation, do not mandate evaluation for encephalitis. The major requirement for at least 24 h of altered mentation was selected to exclude the post-ictal state seen in patients with febrile seizures.  ^d^ CSF pleocytosis is suggestive of an inflammatory process of the brain parenchyma, meninges, or both (meningoencephalitis). The absence of CSF pleocytosis, however, does not exclude encephalitis. It is recognized that the CSF may be devoid of cells in immunocompromised patients (Fodor et al., Neurology 1998 51:554–59) or early in the course of infection (Weil et al, Clin Infect Dis 2002 34:1154–57; Mook-Kanamori et al., J Am Geriatr Soc 57:1514–15; Jakob et al., Crit Care Med 2012 40:1304–8). Conversely, the CSF profile with inflammation limited to the meninges may be indistinguishable from that in patients with encephalitis. In most cases of encephalitis, however, the absolute number of leukocytes is <1000/mm3 and lymphocytes typically predominate. To ensure adequate sensitivity of the definition, the group defined CSF pleocytosis as ≥5 WBC/mm3.  In cases where there are large numbers of red blood cells in the CSF, such as with a traumatic lumbar puncture, the following formula may allow correction of the WBC count: True CSF WBC = actual CSF WBC (WBC in blood X RBC in CSF)/RBC in blood (Tunkel A. In Mandel ed., Principles and Practice of Infectious Diseases, 7th ed., 2010:1183–88; Bonadio Pediatr Infect Dis J 1992 11:423–31).  Notably, rules for adjusting leukocytes in blood-contaminated CSF have not been well validated (Bonsu and Harper, Pediatr Infect Dis J 2006 25:8–11).  ^e^ Neuroimaging plays a crucial role in the evaluation of patients with suspected encephalitis, as it may support the diagnosis of a specific etiology or identify alternate conditions that mimic encephalitis. Magnetic resonance imaging (MRI) is the radiologic modality of choice for evaluation of patients with suspected encephalitis. Multiple studies have confirmed MRI to be superior to computed tomographic (CT) scanning for demonstration of CNS abnormalities (Tunkel et al. Clin Infect Dis 2008 47:303–27; Glaser et al. Clin Infect Dis 2006 43:1565–77). MRI may aid in defining an etiology, as localization of inflammation may be suggestive of particular pathogens (eg, temporal lobe involvement in patients with herpes simplex virus encephalitis) or of an autoimmune phenomenon (eg, demyelination in patients with acute disseminated encephalomyelitis). A noncontrast CT scan is most useful in evaluating safety in the performance of a lumbar puncture and in excluding alternative diagnoses such as subarachnoid hemorrhage. We recognize that MRI or CT may not be available in resource-limited settings, in which case the diagnosis of encephalitis will need to rely on clinical and laboratory criteria.  ^f^ EEG abnormalities reported in cases of encephalitis range from nonspecific generalized slowing to distinctive patterns suggestive of specific entities, including repetitive sharp wave complexes over the temporal lobes or periodic lateralizing epileptiform discharges in HSV-1 (Lai and Gragasin J Clin Neurophysiol 1988 5:87–103) and bilateral synchronous periodic sharp and slow waves associated with subacute sclerosing panencephalitis (Gutierrez et al. Dev Med Child Neurol 2010 52:901–7). EEG abnormalities are frequently nonspecific and may be attributable to medications or metabolic abnormalities. The EEG may identify epileptiform discharges in the absence of clinical evidence of seizure activity (subclinical or nonconvulsive status epilepticus) as a cause of obtundation. |
| Abbreviations: CNS. central nervous system; CSF. cerebral spinal fluid; EEG. electroencephalogram; RBC. red blood cell; WBC. white blood cell. |
| ^1^Venkatesan A. Tunkel AR. Bloch KC. Lauring AS. Sejvar J. Bitnun A. et al. Case definitions. diagnostic algorithms. and priorities in encephalitis: consensus statement of the international encephalitis consortium. Clin Infect Dis. 2013;57: 1114–1128. https://doi.org/10.1093/cid/cit458 |

**S2 Table:** Clinical and demographic data for the Development Cohort (DC) part 1

| 1 Jan 2012 - 30 Jun 2019 | | | **NS Infection** | | **No NS Infection** | | **Statistics** | |
| --- | --- | --- | --- | --- | --- | --- | --- | --- |
| **Sample** | 783 | 100% | 97 | 12.39% | 686 | 87.61% |  |  |
| **Variables** | n | % | **T+** | **T-** | **T+** | **T-** | **χ^2^** | **p-value*** |
| Sex (male) | 458 | 58.5 | 70 | 27 | 388 | 298 | 8.5 | **0.0035** |
| HIV/AIDS | 428 | 54.7 | 79 | 18 | 349 | 337 | 32 | **< 0.0001** |
| Encephalopathy^1^ | 296 | 37.8 | 75 | 22 | 221 | 465 | 73.43 | **< 0.0001** |
| Fever | 94 | 12 | 21 | 76 | 73 | 613 | 9.736 | **0.0018** |
| Focal Deficit^1^ | 17 | 2.2 | 11 | 86 | 6 | 680 | 43.76 | **< 0.0001** |
| Seizures^1^ | 43 | 5.5 | 25 | 72 | 18 | 668 | 87.63 | **< 0.0001** |
| ICU death | 206 | 26.3 | 26 | 71 | 180 | 506 | 0.014 | 0.9059 |
| Hospital death | 266 | 34 | 38 | 59 | 228 | 458 | 1.33 | 0.2480 |

^1^Neurological reasons for intensive care unit (ICU) admission. as defined by SAPS (Simplified Acute Physiology Score) III. *Bold: p<0.1 was considered to indicate statistical significance.

NS: nervous system; T+: positive criteria result; T-: negative criteria result; χ^2^: chi-squared test; Aids: acquired immune deficiency syndrome; Fever: temperature equal or above 38 degrees Celsius or 100.4 degrees Fahrenheit; Encephalopathy: vigilance disturbances. confusion. agitation. delirium. coma. stupor. obtunded patient; Focal deficit: hemiplegia. paraplegia. tetraplegia.

**S3 Table:** Clinical and Demographic data of the Development Cohort (DC) part 2

| 1 Jan 2012 - 30 Jun 2019 | | | **NS Infection** | | **No NS Infection** | | **Statistics** | |
| --- | --- | --- | --- | --- | --- | --- | --- | --- |
| Total | 783 patients | | 97 | 12.39% | 686 | 87.61% |  |  |
| **Variables** | **median** | **IQR** | **median** | **IQR** | **median** | **IQR** | **U (CI)** | **p-value*** |
| Age (years) | 48 | 46-49 | 42 | 33-51 | 49 | 37-62 | -7 (-10 - -4) | **< 0.0001** |
| CSF WBC (/mm^3^) | 0 | (0-27) | 4 | 0-13 | 0 | (0-0) | 4 (3-5) | **<0.0001** |
| GCS (points) | 15 | 14-15 | 11 | 8-14 | 15 | 14-15 | -3 (-3 - -2) | **< 0.0001** |
| Hospital time (days) | 14 | 13-15 | 26 | 12.75 - 50.5 | 13 | 6-28 | 10 (6-14) | **< 0.0001** |
| Time before ICU  (days) | 1 | 0-1 | 2 | 0 - 11.75 | 1 | 0 - 4 | 0 (0-1) | **0.0285** |
| ICU time (days) | 6 | 5-6.4 | 10 | 5-15.25 | 5 | 3-11 | 3 (2-5) | **< 0.0001** |
| SAPS 3 (points) | 56 | 47-67 | 59.5 | 47-70.5 | 56 | 47-66.25 | 3 (-1-7) | 0.1954 |
| SOFA (points) | 5 | 4-5 | 5 | 2-9 | 5 | 2-9 | 0 (-1 - 1) | 0.56 |

*bold: p<0.05 was considered to indicate statistical significance. ** worst value calculated in the first hour of ICU admission.

NS: nervous system. IQR: interquartile range. U: Mann-Whitney test. CI: 95% confidence interval. CSF: cerebrospinal fluid. WBC: white blood cell count/mm^3^. Time before ICU: length of hospital stay before admission to the ICU. ICU: intensive care unit. SAPS III: Simplified Acute Physiology Score III. SOFA: Sequential Organ Failure Assessment score. GCS: lowest Glasgow Coma Scale.

**S4 Table.** Central nervous system infections - LASSO regression stepwise equation - Development Cohort (DC)

| **Variables** | **Coefficient** | **Std. Error** | **t** | **p-value** | **Partial correlation** | **Collinearity statistics** | |
| --- | --- | --- | --- | --- | --- | --- | --- |
|  |  |  |  |  |  | **Tolerance** | **VIF** |
| ***(Constant)*** | ***-0.0766*** | ***0.009*** | ***8.777*** | ***<0.0001*** |  |  |  |
| CSF WBC >2 cells/mm^3^ | 0.7289 | 0.04077 | 17.878 | **<0.0001** | 0.587 | 0.915 | 1.093 |
| Age <56 (years) | 0.05849 | 0.02093 | 2.794 | **0.0053** | 0.128 | 0.850 | 1.176 |
| Sex (Male) | 0.02515 | 0.01763 | 1.426 | 0.1542 | 0.115 | 0.958 | 1.044 |
| Encephalopathy | 0.04881 | 0.02144 | 2.277 | **0.0231** | 0.346 | 0.627 | 1.594 |
| Fever | 0.07189 | 0.02632 | 2.731 | **0.0065** | 0.107 | 0.986 | 1.014 |
| Focal Neurologic Deficit | 0.3104 | 0.05882 | 5.277 | **<0.0001** | 0.242 | 0.963 | 1.039 |
| GCS<14 (points) | 0.1075 | 0.02418 | 4.444 | **<0.0001** | 0.388 | 0.594 | 1.683 |
| Seizures | 0.2374 | 0.03910 | 6.072 | **<0.0001** | 0.371 | 0.889 | 1.125 |
| ICU time>13 (days) | 0.04371 | 0.02556 | 1.710 | 0.0877 | 0.085 | 0.759 | 1.317 |
| Hospital time >22 (days) | 0.02602 | 0.02215 | 1.175 | 0.2405 | 0.197 | 0.646 | 1.547 |
| Time Before ICU >5 (days) | 0.02065 | 0.01891 | 1.092 | 0.2751 | 0.074 | 0.868 | 1.152 |
| HIV/AIDS | 0.01055 | 0.02027 | 0.521 | **0.0304** | 0.237 | 0.797 | 1.255 |
| - 1. **Analysis of Variance** | | | | **Model summary** | | | |
| **Source** | **DF** | **Sum of**  **Squares** | **Mean**  **Square** | **Coefficient of determination R2** | | | 0.513 |
| **Regression** | 12 | 50.661 | 4.222 | **R2-adjusted** | | | 0.507 |
| **Residual** | 769 | 42.7832 | 0.05563 | **Multiple correlation**  **coefficient** | | | 0.716 |
| **Z** | 81.924 | | p<0.0001 | **Residual standard deviation** | | | 0.323 |

*p<0.05 was considered to indicate statistical significance. LASSO: least absolute shrinkage and selection operator. VIF: Variable Inflation Factors. NS: nervous system. CSF: cerebrospinal fluid. WBC: white blood cell count/mm^3^. ICU: intensive care unit. Encephalopathy: any altered consciousness - coma. stupor. obtundation or delirium. Fever: temperature equal or above 38 degrees Celsius or 100.4 degrees Fahrenheit. GCS: Glasgow Coma Scale. Time before ICU: length of hospital stay before admission to the ICU.

**S5 Table.** Central nervous system infection (CNSI) profile - development plus internal validation cohorts, external validation cohort

| **Development and validation cohort 1 - 112** | | | **Validation cohort 2 - 88** | | |
| --- | --- | --- | --- | --- | --- |
| **HIV-negative (n=19)** | | | Post surgical CNSI | 45 | 51.13% |
| Meningococcal meningitis | 4 | 21% | Bacterial meningoencephalitis | 14 | 16% |
| Viral meningoencephalitis | 4 | 21% | Viral meningoencephalitis | 14 | 16% |
| Cryptococcosis | 3 | 15.78% | Asseptic encephalitis | 5 | 5.7% |
| Tuberculosis | 3 | 15.78% | Bacterial brain abscess | 3 | 3.4% |
| Paracoccidioidomycosis | 1 | 5.26% | Epidural abscess | 2 | 2.27% |
| Pneumococcal meningitis | 1 | 5.26% | WNV meningoencephalitis | 1 | 1.13% |
| *Scedosporium* sp. abscess | 1 | 5.26% | Fungal abscess | 1 | 1.13% |
| Septic emboli | 1 | 5.26% | Syphilis | 1 | 1.13% |
| Syphilis | 1 | 5.26% |  |  |  |
| **HIV-positive (n=93)** | | |  |  |  |
| Cryptococcosis | 26 | 27.95 % |  |  |  |
| Toxoplasmosis | 22 | 23.65% |  |  |  |
| Tuberculosis | 16 | 17.20% |  |  |  |
| PML | 12 | 13% |  |  |  |
| CMV | 6 | 6.45% |  |  |  |
| Herpes simplex encephalitis | 2 | 2.16% |  |  |  |
| Histoplasmosis | 1 | 1.08% |  |  |  |
| HIV encephalitis | 1 | 1.08% |  |  |  |
| Meningococcal meningitis | 1 | 1.08% |  |  |  |
| Pneumococcal meningitis | 1 | 1.08% |  |  |  |
| Septic emboli | 1 | 1.08% |  |  |  |
| Syphilis | 1 | 1.08% |  |  |  |
| Sporotrichosis | 1 | 1.08% |  |  |  |
| Varicella-zoster encephalitis | 1 | 1.08% |  |  |  |
| Viral meningoencephalitis | 1 | 1.08% |  |  |  |

Rio, Brazil: 112 infections / 946 patients. Chicago, USA: 88 / 7,270 patients. HIV: Human immunodeficiency virus; PML: progressive multifocal leukoencephalopathy by JC virus; CMV: cytomegalovirus encephalitis; Septic emboli complicating acute endocarditis. WNV: West Nile Virus

| **S1 Fig.** Calibration slopes of the Development Cohort (DC), Validation Cohort (VC)1 and Validation Cohort 2 |
| --- |
| 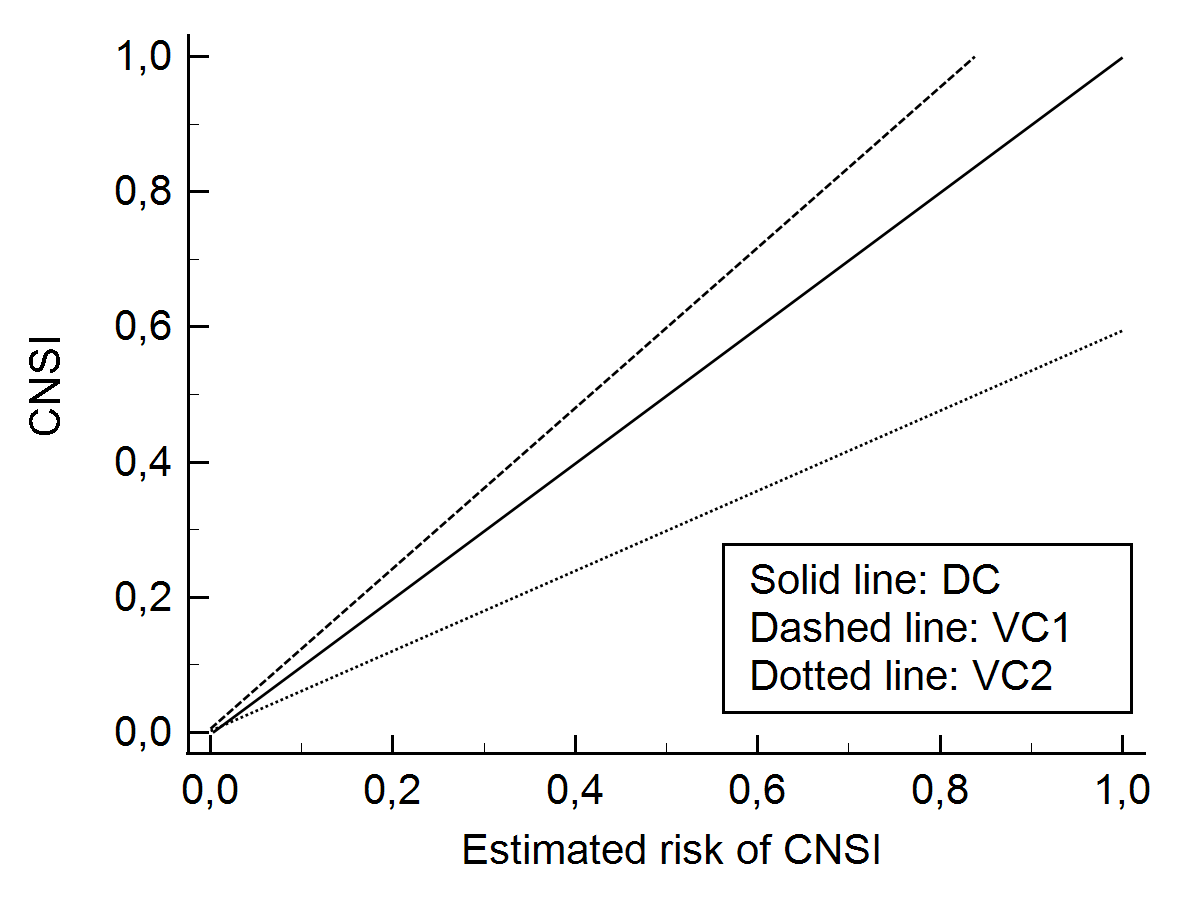 |
| **DC:** y = -0.002583 (-0.02065 to 0.01548 CI; P=0.7790) + 1.0013 x (0.9343 to 1.0683; P<0.0001) x  **VC1:** y = 0.005774 (-0.02433 to 0.03588; P=0.7054) + 1.1864 (1.0386 to 1.3343; P<0.0001) x  -Slope Difference to DC: -0.1852; STE 0.09659; P=0.05554  -Intercept difference to DC: -0.02298; STE 0.02045; P=0.2613  **VC2**: y = 0.002511 (-0.02846 to 0.03348; P=0.8735) + 0.5921 (0.5154 to 0.6688; P<0.0001) x  -Slope Difference to DC: 0.4092, STE 0.05191, t=7.8815. DF 1369. P=6.5533.  -Intercept difference to DC: -0.06771. STE 0.01883. t=3.5952, DF 1369. P=0.00033  **CNSI:** central nervous system infection  The pooled data regression equation is y = 0,003339 (-0,01209 to 0,01877, P=0,6713) + 0,7586 (0,7108 to 0,8065, P<0.0001) x with a coefficient of determination R2 of 0.3870 and the residual standard deviation of 0.2637 |
